# Development of a Pipeline for Adverse Drug Reaction Identification in Clinical Notes: Word Embedding Models and String Matching

**DOI:** 10.2196/31063

**Published:** 2022-01-25

**Authors:** Klaske R Siegersma, Maxime Evers, Sophie H Bots, Floor Groepenhoff, Yolande Appelman, Leonard Hofstra, Igor I Tulevski, G Aernout Somsen, Hester M den Ruijter, Marco Spruit, N Charlotte Onland-Moret

**Affiliations:** 1 Laboratory of Experimental Cardiology University Medical Center Utrecht Utrecht University Utrecht Netherlands; 2 Department of Cardiology Amsterdam University Medical Centers, VU University Medical Center Amsterdam Netherlands; 3 Central Diagnostic Laboratory University Medical Center Utrecht Utrecht University Utrecht Netherlands; 4 Cardiology Centers of the Netherlands Utrecht Netherlands; 5 Department of Public Health and Primary Care Leiden University Medical Center Leiden University Leiden Netherlands; 6 Leiden Institute of Advanced Computer Science Leiden University Leiden Netherlands; 7 Department of Epidemiology Julius Center for Health Sciences and Primary Care University Medical Center Utrecht, Utrecht University Utrecht Netherlands

**Keywords:** adverse drug reactions, word embeddings, clinical notes

## Abstract

**Background:**

Knowledge about adverse drug reactions (ADRs) in the population is limited because of underreporting, which hampers surveillance and assessment of drug safety. Therefore, gathering accurate information that can be retrieved from clinical notes about the incidence of ADRs is of great relevance. However, manual labeling of these notes is time-consuming, and automatization can improve the use of free-text clinical notes for the identification of ADRs. Furthermore, tools for language processing in languages other than English are not widely available.

**Objective:**

The aim of this study is to design and evaluate a method for automatic extraction of medication and Adverse Drug Reaction Identification in Clinical Notes (ADRIN).

**Methods:**

Dutch free-text clinical notes (N=277,398) and medication registrations (N=499,435) from the Cardiology Centers of the Netherlands database were used. All clinical notes were used to develop word embedding models. Vector representations of word embedding models and string matching with a medical dictionary (Medical Dictionary for Regulatory Activities [MedDRA]) were used for identification of ADRs and medication in a test set of clinical notes that were manually labeled. Several settings, including search area and punctuation, could be adjusted in the prototype to evaluate the optimal version of the prototype.

**Results:**

The ADRIN method was evaluated using a test set of 988 clinical notes written on the stop date of a drug. Multiple versions of the prototype were evaluated for a variety of tasks. Binary classification of ADR presence achieved the highest accuracy of 0.84. Reduced search area and inclusion of punctuation improved performance, whereas incorporation of the MedDRA did not improve the performance of the pipeline.

**Conclusions:**

The ADRIN method and prototype are effective in recognizing ADRs in Dutch clinical notes from cardiac diagnostic screening centers. Surprisingly, incorporation of the MedDRA did not result in improved identification on top of word embedding models. The implementation of the ADRIN tool may help increase the identification of ADRs, resulting in better care and saving substantial health care costs.

## Introduction

### Background

Literature shows that adverse drug events (ADEs) and, more specifically, adverse drug reactions (ADRs) are structurally underreported [1]. Clinical trials may underreport or miss ADRs for various reasons, such as a follow-up that is usually too short to catch long-term effects [2]. In addition, the study population may be healthier or otherwise different from the target population in regular care [3]. As a result, the ADR risk of clinically relevant subgroups such as women and older adults remains unknown [4], which places a societal and economic burden on our health care system. The prevalence of hospital admissions associated with ADRs is reported to be as high as 5.3% and estimated to be twice as high in the older adult population [5]. In the United States alone, ADRs are estimated to generate US $30 billion in unnecessary costs [6]. Efforts have been made to structurally collect information on ADRs both on a national (eg, Lareb in the Netherlands) and international (EudraVigilance [7]) level; however, these pharmacovigilance databases do not include relevant patient characteristics and information about prescription rates.

Regular care data extracted from electronic health records can help in postmarketing surveillance of medication. ADRs are usually not reported in the electronic health record in a structured way, but the clinical notes made during consultations between patients and their physicians may hold relevant information when patients experience an ADR. However, these notes are often stored as free text and thus cannot be easily analyzed [8]. Methods that extract ADRs from these free-text fields are needed to access the full potential of these data.

Natural language processing (NLP) techniques can aid in the differentiation of relevant features from idle free text and prepare free text for research purposes [9,10]. One of the widespread topics in NLP is the use of word embeddings—a vector representation of a text, often established through evaluation of the word’s context. The use of word embeddings for the evaluation of clinical free text for research purposes is increasing [11]. Research has shown that training word embedding models on a domain-specific data set generates better results than training on a general data set [12,13]. As a result, applications of word embedding models are studied in a wide range of topics within the health care domain (eg, evaluation of radiology reports [14], identification of ICD-10 codes [15], and identification of ADEs in English electronic health records [16]) and can potentially be a solution to extract ADRs from Dutch clinical notes.

### Objectives

The objective of this research is to design a method for the identification of ADRs in clinical notes from a regular care database (Adverse Drug Reaction Identification in Clinical Notes [ADRIN]) using unlabeled data and word embeddings. Although the demonstrations in this study have been done with Dutch clinical notes from the cardiovascular domain, the method has been developed in a way that enables generalization not only to other languages but also to other research questions to mine text in clinical notes.

## Methods

### Overview

**Figure 1:**
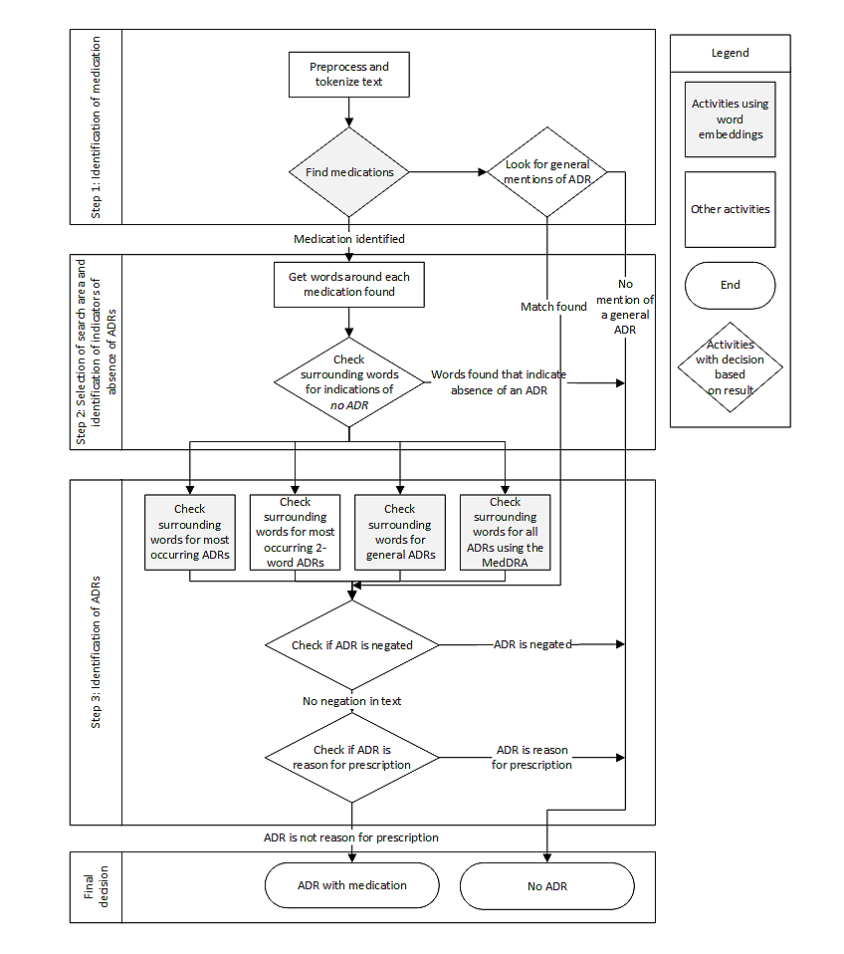
Overview of the different steps in the Adverse Drug Reaction Identification in Clinical Notes method. ADR: adverse drug reaction; MedDRA: Medical Dictionary for Regulatory Activities.

The ADRIN method is based on the implementation of a medical taxonomy to enhance standardized terminology (the Medical Dictionary for Regulatory Activities [MedDRA]) [17] and on word embeddings trained on a large database of medical free text. In addition, a prototype was developed and evaluated on labeled Dutch clinical notes to determine the performance of this method. Figure 1 shows the general workflow of the ADRIN method.

This study focused on the identification of ADRs and the corresponding medications. We assumed that patients were compliant with their medication regimen. We defined an ADR as any unwanted event that led to the discontinuation of the prescribed medication. In the following description, clinical notes are defined as the free text written down in the electronic health record by the physician after a patient’s consultation.

### Data Set

The Cardiology Centers of the Netherlands database is a large regular care database from 13 diagnostic cardiac screening centers. In short, this database consists of 109,151 patients who visited one of the outpatient cardiac screening centers between 2007 and 2018 and includes patient characteristics and information about diagnostic tests [18].

In total, there were 277,398 clinical notes in the database and 499,435 medication prescriptions. Clinical notes were deidentified using DEDUCE [19]. Medication prescriptions contain information about the prescribed medication, start date and end date (if the medication was discontinued at some point), and reason for discontinuation in free text.

**Figure 2:**
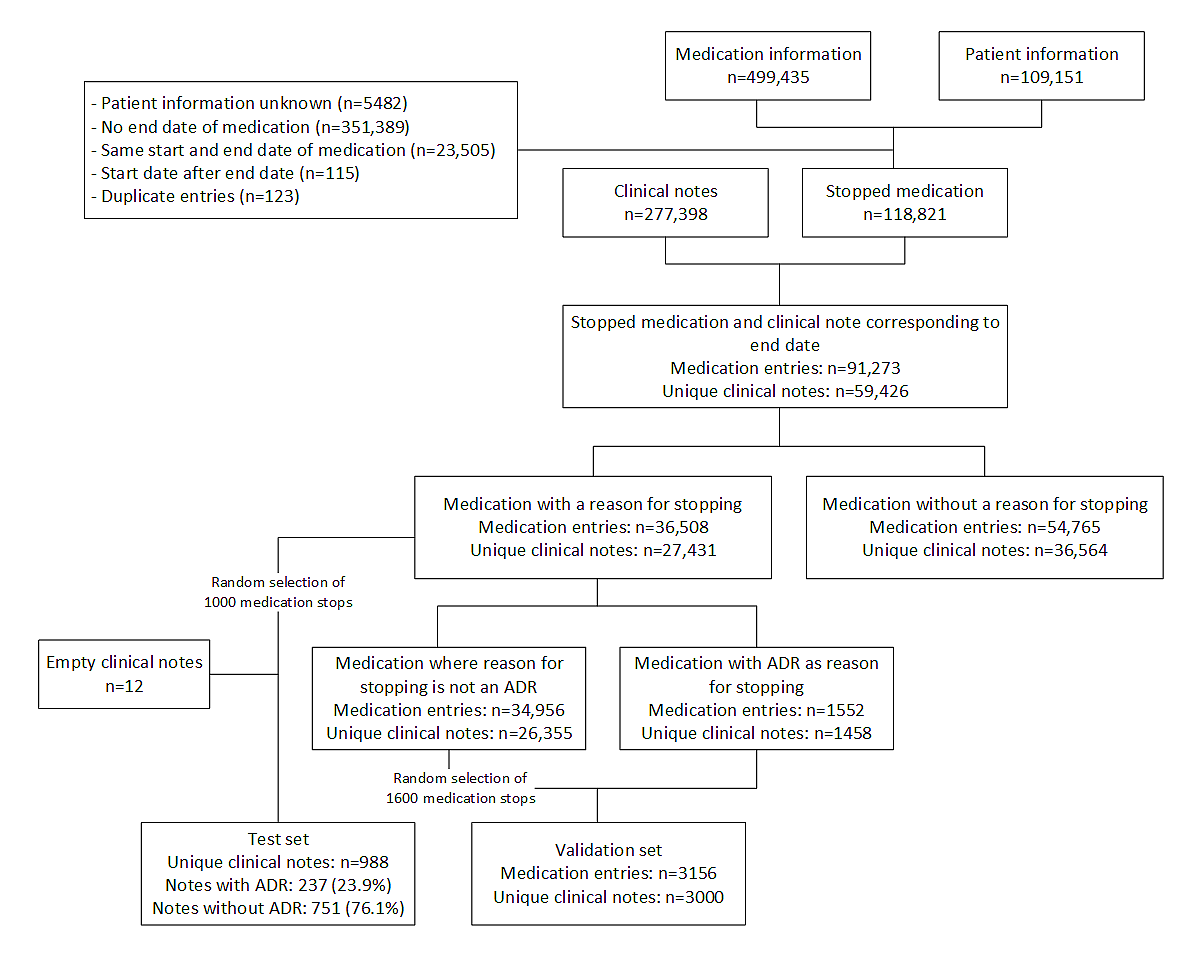
Flowchart of selection of clinical notes and corresponding adverse drug reaction and medication. ADR: adverse drug reaction.

Figure 2 describes the selection of discontinued medication entries from the database. The selected prescriptions were merged with the clinical notes. This resulted in 91,273 discontinued medication entries for which a clinical note was available on the end date of the medication. In cases where multiple prescriptions from the same patient were stopped on the same day (19,992/91,273, 21.9%), the same clinical note was used for all prescriptions. The reason for discontinuation was reported in 40% (36,508/91,273) of the medication prescriptions. From these 91,273 medication entries, we randomly selected 1000 (1.1%) medication entries and corresponding clinical notes as a test set. However, in 1.2% (12/1000) of the cases, the clinical note was empty, resulting in a test set of 988 clinical notes.

The validation set was obtained from discontinued medication entries and consisted of all medication stops with an ADR reported as a reason for discontinuation and a random selection of 1600 medication stops that were not ADR-related. The latter selection was made because we expected that the clinical notes corresponding to these medication stops might also contain information on possible ADRs. Thus, this selection made it more likely that medication and ADRs would be identified when compared with a random selection of all clinical notes (Figure 2). These 2 selections of medication stops were merged with the corresponding clinical notes and resulted in a data set of 3000 unique clinical notes as there were some notes linked to medication stops that reported ADRs as well as medication stops that did not report an ADR.

The Medical Research Ethics Committee of the University Medical Center Utrecht declared that research within the Cardiology Centers of the Netherlands database does not fall under the Dutch Medical Research Involving Human Subjects Act (proposal number 17/359).

### Labeling

In total, 2 researchers (KRS and ME) independently labeled all clinical notes in the test set. Clinical notes containing ADR information were labeled as positive. When a note was labeled positively, all words in the text describing the medication and ADR combinations were extracted. Discrepancies in labeling between the 2 researchers were discussed, and interobserver variability was evaluated. Furthermore, a validation data set of 3000 unique clinical notes was labeled by one of the researchers (either KRS or ME). These notes were used for identification of thresholds for the word embedding models and for intermediate, qualitative, and direct feedback.

### Preprocessing Clinical Notes

Before applying word embedding models to the clinical notes, the text underwent multiple preprocessing steps. First, all text was converted to lowercase and unidecoded. Second, the clinical notes were tokenized with a regular expression tokenizer set to greedy tokenization for every word in the presented text. Third, all numerical tokens were converted into their written form (number normalization [20]). It is assumed that this results in numbers being more closely related in vector space (ie, *16* and *18* vs *sixteen* and *eighteen*). Doses were removed from the text using regular expressions. The doses were removed to reduce the similarity between frequently prescribed doses and specific medications. This would otherwise contaminate the word embedding models used for identification of medication. Finally, for each token, a check was performed to determine if the token was in the unigram word embedding model. If this was not the case, the word was removed from the list of tokens. An example of a text going through this process is presented in Multimedia Appendix 1, Figure S1. The text was preprocessed using Python version 3.7.9 (Python Software Foundation [21]) using the nltk package (version 3.5) [22].

### Word Embedding Models

For the automatic identification of ADRs from the text, word embedding models were developed. In total, 2 Word2Vec models imported from the Python Gensim package (version 3.8.0) [23] were trained on the complete set of 277,398 clinical notes [24]. A unigram model was developed using vectors for single words. This model included all words and derived vectors that occurred more than once in the complete set of clinical notes. The second model used a combination of single words, bigrams, and the derived vectors (bigram model). For the development of this model, words that occurred together >5 times were represented as a vector. Stop words imported from the nltk package [22] were removed from the text. A skipgram approach was used.

The Word2Vec settings were a vector size of 200 dimensions, a window of 5 words around the main word, and 5 iterations of learning. Word embedding models were qualitatively evaluated through inspection of the similarity among words [25].

### Identification of Medication and ADRs

A list of search words was created for both medication and ADRs. The medication search list was based on different groups of cardiovascular medications (Multimedia Appendix 2, Table S1). For ADR identification, the most frequently reported ADRs (Multimedia Appendix 2, Table S2) in the discontinued medication entries were considered. From these ADRs, a list of search words for ADR recognition was compiled (Multimedia Appendix 2, Table S1).

Word embeddings were used for evaluation of the clinical note. First, the cosine similarity between each word in the clinical note and the search words for medication was calculated. A medication was identified if the cosine similarity was above a predefined threshold (Multimedia Appendix 2, Table S1). If no medication was found in the text, a second search was performed to identify a mention of ADRs using more general search words such as *adverse drug reaction*. If these search words were also not identified in the text, the clinical note was automatically labeled as not containing an ADR (Figure 1, step 1).

Second, after identification of a medication, the clinical note was searched for ADRs using a predefined search area around the identified medication (Figure 1, step 2). This search area was restricted to prevent an increasing number of false positives and could be adjusted if it seemed too strict or too wide. This was one of the settings adjusted during the evaluation of the pipeline.

After this, the area was checked for *non-ADR keywords*. These words occurred immediately before or after the medication and indicated a medication change or extension, such as *increase* and *double*. Therefore, these words did not indicate the presence of an ADR. List comparison was used, in which the tokenized form of the clinical note was compared with a list of words that pointed toward a medication change not likely because of an ADR (Multimedia Appendix 2, Table S3).

The final step in the search for ADRs was the actual identification (Figure 1, step 3). In total, 2 sequential approaches were developed for this purpose. The first approach included the application of the MedDRA. A selection of the lower-level MedDRA terms (Lowest Level Terms) [17] was checked with text retrieval and string matching in the defined search area around the medication. Inclusion or exclusion of the MedDRA was one of the settings adjusted during the evaluation of the pipeline.

The second approach for identification of ADRs was the use of unigram and bigram word embedding models. For each word in the search area, the cosine similarity with the search words for ADRs was computed (Multimedia Appendix 2, Table S1). If this similarity was above the predefined threshold, the word was identified as an ADR. Threshold-setting was performed using a grid search. Visual inspection of the graphical representation of the number of correct matches for a specific word (Multimedia Appendix 1, Figure S2) and evaluation of the included words after inspection of the list of most similar words resulted in the setting of the thresholds. For example, in the case of a specific medication, the threshold was set such that spelling mistakes and closely related medications were selected but not words that were related to a significant other medication group or words that did not describe medication but a certain disease or condition. For this analysis, the validation data set was used. This is explained in more detail in Multimedia Appendix 1.

### Pipeline Versions and Tasks

**Figure 3:**
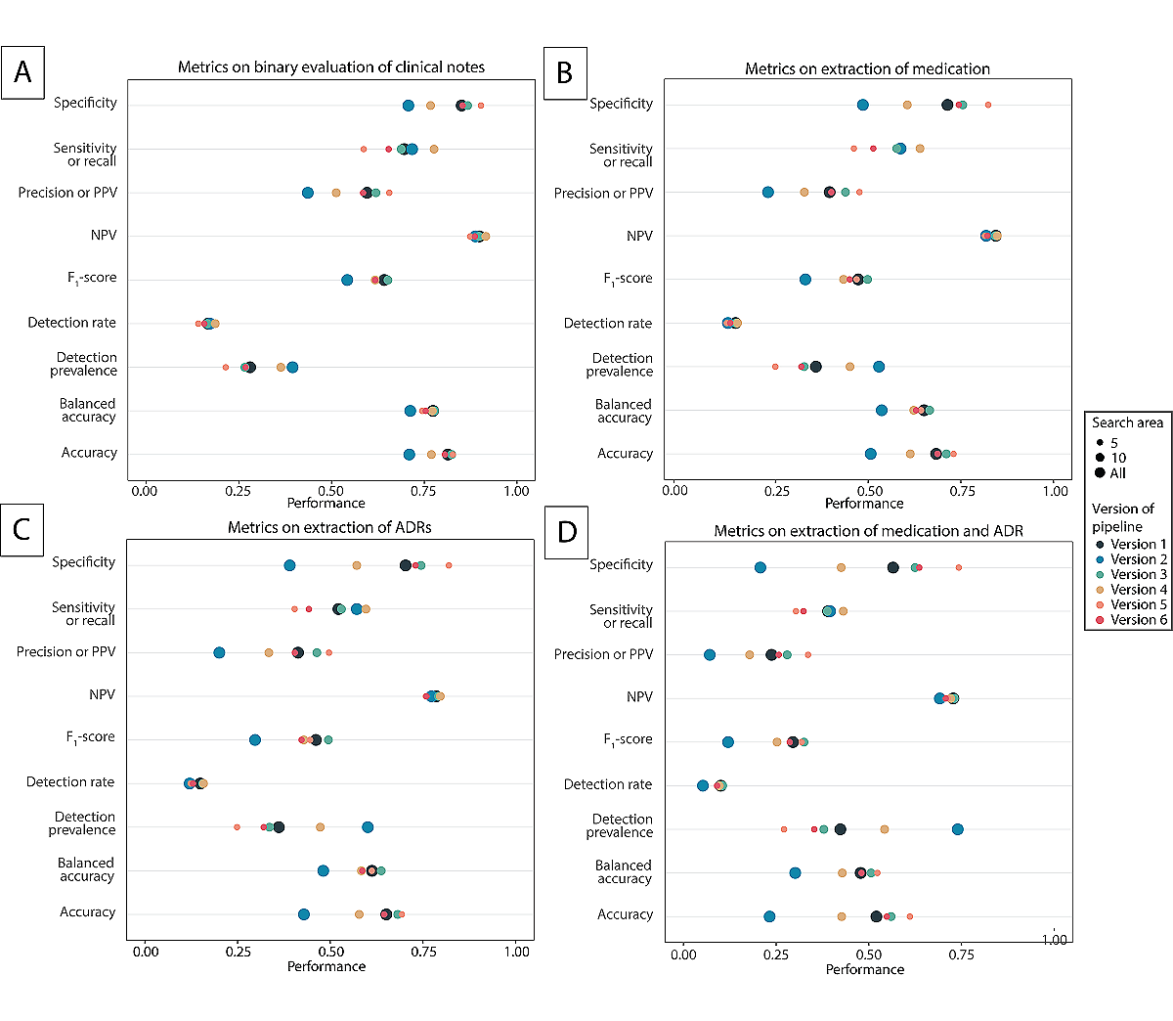
Performance of different experimental versions of the pipeline with the inclusion of the MedDRA on the different tasks (A: binary evaluation, B: medication identification, C: ADR identification, D: medication and ADR + adverse drug reaction identification). ADR: adverse drug reaction; MedDRA: Medical Dictionary for Regulatory Activities; NPV: negative predictive value; PPV: positive predictive value.

**Figure 4:**
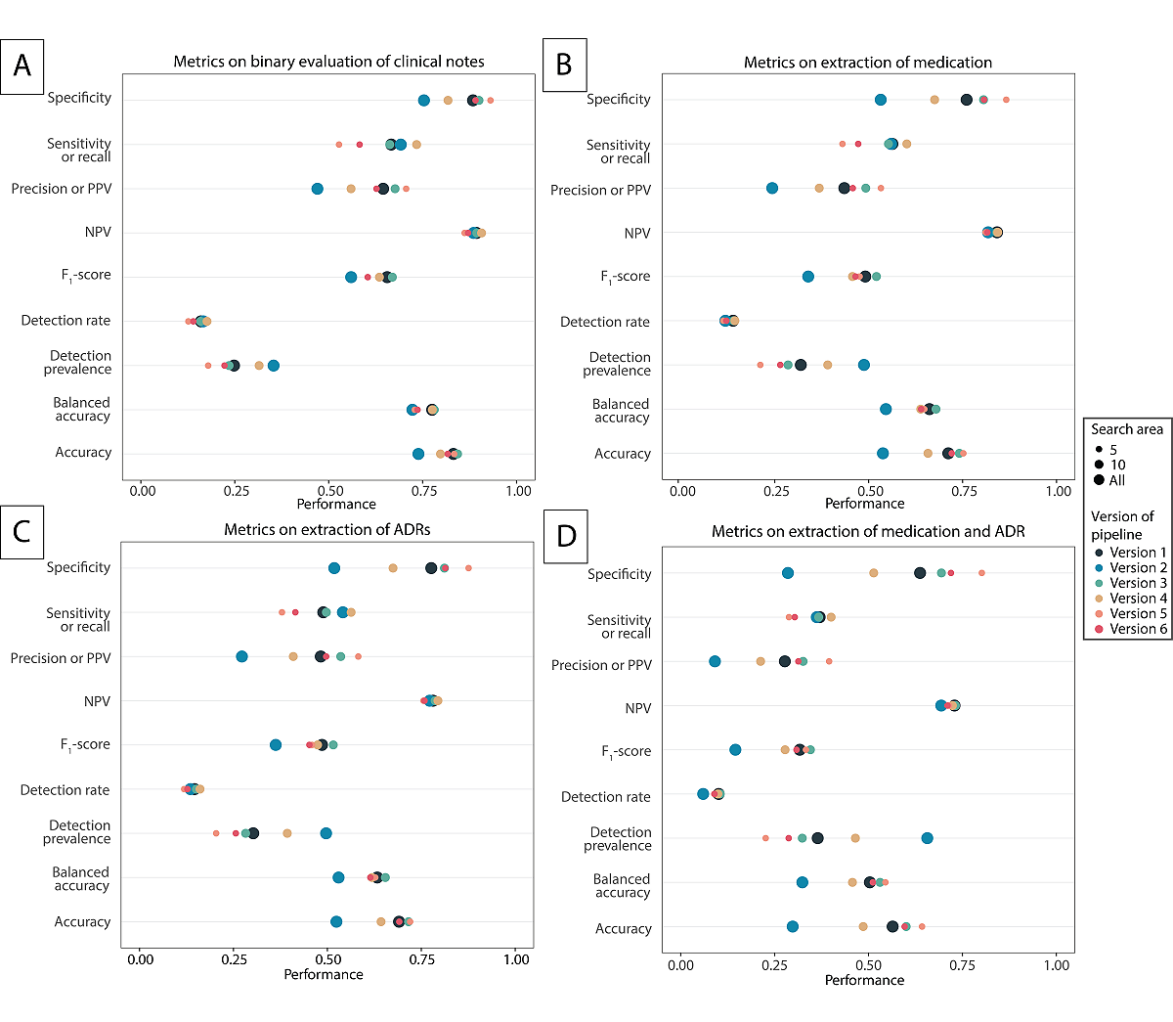
Performance of different experimental versions of the pipeline without the use of the MedDRA on the different tasks (A: binary evaluation, B: medication identification, C: ADR identification, D: medication and ADR + adverse drug reaction identification). ADR: adverse drug reaction; MedDRA: Medical Dictionary for Regulatory Activities; NPV: negative predictive value; PPV: positive predictive value.

The pipeline was developed to execute four different tasks: (1) a binary classification of whether the clinical note contained an ADR (Figure 3A and Figure 4A), (2) the extraction of the medication that causes an ADR (Figure 3B and Figure 4B), (3) the extraction of the ADR individually (Figure 3C and Figure 4C), and (4) the exact extraction of the medication and corresponding ADR (Figure 3D and Figure 4D).

Multiple settings were changed during the analysis to evaluate the performance of the predefined tasks of different experimental designs of the pipeline: inclusion or exclusion of the MedDRA for ADR identification, inclusion or neglect of punctuation for demarcation of the search area, and size of the search area. Table 1 provides an overview of the different settings evaluated in the versions of the pipeline. Analysis of the pipeline was performed using Python version 3.7.9 [21].

**Table 1:** Settings of the pipeline features of the different computational experiments.

Version	Words in search area	Considering punctuation	Version without MedDRA^a^
1A	All	Yes	1B
2A	All	No	2B
3A	10	Yes	3B
4A	10	No	4B
5A	5	Yes	5B
6A	5	No	6B

^a^MedDRA: Medical Dictionary for Regulatory Activities.

### Performance Metrics

The pipeline was evaluated on the test set of 988 labeled clinical notes. Different metrics were calculated to assess the performance of different versions of the pipeline. The metrics that were calculated included accuracy and balanced accuracy, sensitivity, specificity, precision or positive predictive value, negative predictive value, recall, F_1_ score, detection rate, and detection prevalence. An elaborate overview of the performance metrics and the evaluation process can be found in Multimedia Appendix 3, Table S1 and Tables S2-S6, respectively. The outcome was evaluated using the R programming language version 4.0.2 (R Foundation for Statistical Computing [26]) and RStudio version 1.3.1093 (RStudio Team [27]). The caret package was used for evaluation (version 6.0-86) [28].

## Results

### Data Set

The information on the complete data set for word embedding models, validation set, and test set is described in Table 2. The characteristics of the included free text are the informal writing style, use of abbreviations, and relatively short text length. Multimedia Appendix 3 contains 4 different translated examples of clinical notes, as shown in Multimedia Appendix 3, Table S2.

**Table 2:** Characteristics of selected clinical notes for development of the word embedding models, validation set, and test set.

Variable	Word embedding models	Validation set	Test set
Language	Dutch	Dutch	Dutch
Number of unique records	277,398	3000	988
Unique patients	108,940	2707	955
Number of unique tokens	96,086	9297	5464
Number of tokens per record, mean (SD)	54 (44)	53 (44)	53 (48)
Number of tokens per record, median (IQR)	43 (26-70)	42 (25-67)	41 (24-66)
Individuals of the female sex, n (%)	56,527 (51.89)	1320 (49.07)	459 (48.06)

### Word Embedding Models

Several search terms of the prototype were independently reviewed in the word embedding models to evaluate the performance of the word embedding models. Table 3 lists a selection of these keywords and the 5 most similar words. It was noted that, if the search word was a specific group of medications (eg, β-blockers), other groups of medications were also identified (eg, *diltiazem* in the case of the search word β*-blocker*). As the identified word was used for the analysis and not the search word, this had no consequences for the analysis.

Free text from clinical notes was used in the training of the word embedding models. These are domain-specific data, which can improve the embedding of domain-specific words. An illustrative example is the word embedding of *red*. In our word embedding models trained specifically on medical text, *red* was closely associated with *itching*, *swollen*, *irritated*, and *colourings*, whereas, in word embeddings on general text, *red* would be associated with other colors.

**Table 3:** Selection of results from the word embedding models, adverse drug reaction, and medication search words, and a selection of the most relevant similar words where spelling mistakes are excluded. Similarity is based on the cosine similarity.

Keyword	Most similar words in Dutch (English, cosine similarity)
*Pijn op de borst* (chest pain)	*Druk op de borst* (chest pressure, 0.80), *kramp op de borst* (chest cramping, 0.70), *pijn in de armen* (pain in the arms, 0.68), and *retrosternale pijn* (retrosternal pain, 0.67)
*Verminderde conditie* (decreased condition)	*Afname conditie* (decreasing stamina, 0.63), *conditieverlies* (loss of condition, 0.63), *verminderde inspanningstolerantie* (decreased exercise tolerance, 0.62), and *overmating transpireren* (excessive sweating, 0.62)
*Oedeem* (edema)	*Perifeer* (peripheral edema, 0.81), *enkeloedeem* (ankle edema, 0.80), *pitting* (pitting edema, 0.80), and *enkels* (ankle edema, 0.75)
*Hoesten* (coughing)	*Sputum* (sputum, 0.75), *slijm* (mucus, 0.71), *hoestklachten* (coughing complaints, 0.70), and *kuchen* (to cough, 0.70)
*Duizelig* (dizziness)	*Zweterig* (sweaty, 0.73), *misselijk* (nauseous, 0.71), *zweverig* (floaty, 0.70), and *draaierig* (dizzy, 0.69)
*Statine* (statin)	*Simvastatine* (simvastatin, 0.80), *pravastatine* (pravastatin, 0.76), *crestor* (rosuvastatin, 0.75), and *atorvastatine* (atorvastatin, 0.74)
*Betablokker* (β-blocker)	Metoprolol (0.74), atenolol (0.71), diltiazem (0.66), and bisoprolol (0.65)
*Antistolling*	*Acenocoumarol* (acenocoumarin, 0.80), *anticoagulantia* (anticoagulants, 0.78), *NOAC* (novel oral anticoagulant, 0.77), and *fenprocoumon* (phenprocoumon, 0.74)
*Amlodipine*	Nifedipine (0.85), lisinopril (0.82), barnidipine (0.81), and enalapril (0.79)

### Interobserver Variability

A test set (n=988 clinical notes) was manually labeled by 2 independent researchers (KRS and ME) and used for the evaluation of the pipeline. During this process, 91.9% (908/988) of the clinical notes were identically labeled. This resulted in an interobserver variability of 91% for the binary presence of an ADR. Regarding the literal extraction of the ADR and the medication, there were 21.8% (215/988) of instances where the result differed among the researchers. This was mostly due to a difference in taking adjectives or adverbs into account or a different interpretation of the clinical note. As the pipeline was trained on 1-word and 2-word ADRs, it was decided that these words would not be considered.

Manual labeling of the 988 clinical notes in the test set resulted in 23.9% (237/988) notes that were binary classified as containing an ADR. In the notes, 286 medication names (task 2) and 364 individual ADRs (task 3) were mentioned. These notes contained a total of 392 combinations of triggered ADRs (task 4) and corresponding medications.

### Evaluation of the Pipeline

Figures 3 and 4 show the performance of the pipeline on the different metrics and for the different tasks. Multimedia Appendix 2, Table S4 shows the values for true and false negatives and true and false positives per version and per task. The task for binary classification achieved the highest accuracy, varying from 0.70 to 0.84 (Figure 3A). However, as this was the easiest task, the accuracy of the pipeline on the exact extraction of medication and ADR together was much lower, varying from 0.23 to 0.64 (Figure 3D).

If we look at the specific settings of the different pipelines, the results show that the addition of the MedDRA to the pipeline did not lead to an increase in the performance of the pipeline (Figures 4A-4D). Overall, the inclusion of punctuation led to a better performance than transcending sentences (versions 1, 3, and 5), and a search area of 5 words seemed to lead to the best results overall (versions 5 and 6).

The negative predictive value—the chance that no ADR was present when the pipeline did not produce an ADR—was approximately the same per task (0.69-0.91) for all versions of the pipeline. However, the positive predictive value (ie, the chance that, when the pipeline reported an ADR, it was in fact reported in the clinical notes) varied much more per version (Figures 3 and 4) and varied between 0.071 and 0.71. This could be explained by the proportion of false negatives. The proportion of false negatives did not vary much per version of the pipeline for a given task. However, the proportion of false positives had much more variety, caused by a change in the search area and the inclusion or exclusion of punctuation, which led to more ADRs found with a specific medication.

The optimal version of the pipeline depends on the task for which the pipeline is used. If the task is to select notes based on whether they contain ADRs, the results of the binary classification task (task 1) are most relevant. For this task, version 3B (ie, no MedDRA used, search area of 10 words, and considering punctuation) generated the highest accuracy (0.84) and F_1_ score (0.67). In this case, 8.1% (80/988) of notes were classified as false negatives, indicating that 8.1% (80/988) of notes would not be selected when looking for ADRs. The most optimal version based on accuracy for identification of medication, ADRs, and ADRs and medication combined was version 5B, with an accuracy for the different tasks of 0.75, 0.72, and 0.64, respectively. Version 3B was the optimal version when emphasis was on the F_1_ score, with scores of 0.52, 0.52, and 0.35 for identification of medication, ADRs, and medication and ADRs combined, respectively.

During the evaluation of the notes in the test set, the prototype incorporating the MedDRA required approximately 70 minutes to generate an outcome for all notes, whereas the versions without the MedDRA took approximately 14 seconds.

## Discussion

### Principal Findings

In this study, the ADRIN method and a corresponding prototype were developed. The method was evaluated on a subset of clinical notes. Different versions of the prototype led to differing results on the various tasks. The optimal version of the pipeline depends on the task and the trade-off being made—Is it more valuable to find as many medication and ADR combinations as possible or to find fewer ADRs but also make fewer mistakes? If the goal is the former, a larger search area is better. However, even with the entire note as the search area, at least 8% of all medication and ADR combinations were missed. When one wants to be more accurate, a smaller search area is preferred, and punctuation should be considered. This reduces the number of false positives generated, which results in increased accuracy and F_1_ score.

Surprisingly, the versions incorporating the MedDRA performed worse on most tasks than the same versions without the MedDRA. The negative effect of the MedDRA on the performance was due to the large increase in false positives it generated. This was caused by string matching with the MedDRA, leading to more identifications than the specific set of frequently occurring ADRs defined by the predefined search words. Incorporation of the MedDRA could lead to an improved uptake of rare ADRs, but this was not evaluated in more detail. Furthermore, misspelled ADRs were not recognized by the MedDRA search, creating added value for the incorporation of word embedding models. Moreover, implementation of the MedDRA in the prototype significantly increased execution time, a significant attribute if real-time evaluation of clinical notes is required.

Illustrative of the underreporting of ADRs is that, in 60% (54,765/91,273) of the discontinued medication entries, no reason was reported for ending the medication in the registration of a patient’s medication. However, 61.5% (36,564/59,426) of clinical notes were matched to these medication entries, which illustrates the potential additional value of clinical notes in unraveling ADRs in this data set.

When we put these results in light of the ongoing developments of ADR extraction from clinical notes, we see that the performance of our pipeline is similar to that of other presented pipelines. First, most publications have focused on the automatic extraction of ADRs, ADEs, or adverse events [29-32], whereas our study identified the combination of medication and triggered ADR. Another publication that identified both ADR and medication showed increased performance, with F_1_ scores for drug, ADR, and combination of drug and ADR of 0.930, 0.887, and 0.534, respectively [33], versus the performance of 0.52, 0.51, and 0.34, respectively, that we showed. When comparing methodologies, our method predominantly relies on internal information and similarity from word embeddings, whereas Tang et al [33] use external reference sources for the development of their dictionaries, which is the case in most studies. The use of word embeddings increases the identification of spelling mistakes in medication and ADRs, brand names, and synonyms. However, in our methodology, there were also an increased number of false positives.

Thus, word embedding models can be used for the identification of spelling mistakes and brand names of medications. However, for the identification of synonyms, the use case must be critically evaluated. It was shown that words that indicated what was done with a specific prescription (eg, *to lower* and *to increase*) were considered similar by the word embedding models. Therefore, it is not suitable to use word embedding models for identification of *non-ADR* keywords, which was solved with string matching in the ADRIN method. The use of domain-specific word embedding models is not new or limited to ADR identification but is increasingly used in the evaluation of clinical notes (eg, in ICD-10 classification [15] and anonymization [34]).

Second, publications on identification of ADRs in the English language are numerous, using different methods such as General Architecture for Text Engineering NLP [35], trigger words [30], or trigger phrases [31]. Regarding foreign languages, the field is maturing. Methods developed for the English language can, in some cases, be transferred to other languages. However, the effort that must be put into this depends on the complexity of the task and the level of text interpretation [36]. For example, a study of Danish clinical notes obtained better performance (recall of 0.75 in [32] vs 0.59 in this study) for sole ADR identification. This study missed approximately one-fourth of all possible ADRs, whereas our optimal performance missed approximately 40%. However, this pipeline included manual dictionary selection and more rule-based filters in the model [32].

We chose to use the presence of a mention of medication in the clinical note as the starting point for identification of an ADR. However, this might result in experienced ADRs being missed. The performance of the pipeline might benefit from the removal of the identification of medication and, for example, coupling with structured medication prescriptions to obtain information about medication use. However, the end user should be aware that this might also increase the number of false positives as the presence of an ADR is no longer limited by the presence of medication.

Limitations that were identified during the evaluation of the method and prototype are primarily related to missed ADRs from the clinical free text even when the entire clinical note was used for analysis. This problem can be solved by lowering the identifying threshold, but this would also lead to a potentially large increase in false positives. The use of machine and deep learning models can improve the performance of the ADRIN method. However, a large data set of labeled clinical notes is required to train machine and deep learning models, which was unavailable during the development of this model.

An overall limitation of the prototype is the direct translatability to other languages. The word embedding models were specifically trained on Dutch clinical notes. Search terms for word embedding functions must be translated into the new language to implement this method in clinical notes in a different language. Moreover, word embedding models must be trained with notes in the specific language before applying the developed method. Therefore, a large number of clinical free-text notes are required. Because of ethical and privacy constraints, this can be hard to acquire. However, it is technically possible to test and validate the ADRIN method in other languages through translation of search words and negations and after training word embedding models with the specific language.

### Conclusions

In conclusion, the ADRIN method and prototype are effective in recognizing ADRs in Dutch clinical notes. Surprisingly, incorporation of the MedDRA did not result in improved identification on top of word embedding models. However, not all versions of the prototype were equally accurate. Different parameter settings can be chosen for the prototype to optimize the task of the model. In a future stage, incorporation of a pipeline in an electronic health record environment can lead to automatic identification and registration of ADRs. This saves the physician’s precious time and decreases the previously mentioned underreporting of ADRs in clinical care, increasing our knowledge about ADRs, which might ultimately benefit the patient.

## Appendix

Multimedia Appendix 1 Supplementary methods: text preprocessing and threshold setting for word embedding models.

Multimedia Appendix 2 Overview of model settings and results.

Multimedia Appendix 3 Evaluation of the pipeline.

## Abbreviations

ADE: adverse drug event
ADR: adverse drug reaction
ADRIN: Adverse Drug Reaction Identification in Clinical Notes
MedDRA: Medical Dictionary for Regulatory Activities
NLP: natural language processing

## References

[1] Hazell L, Shakir SA (2006). Under-reporting of adverse drug reactions : a systematic review. Drug Saf.

[2] Seruga B, Templeton AJ, Badillo FE, Ocana A, Amir E, Tannock IF (2016). Under-reporting of harm in clinical trials. Lancet Oncol.

[3] Leening MJ, Heeringa J, Deckers JW, Franco OH, Hofman A, Witteman JC, Stricker BH (2014). Healthy volunteer effect and cardiovascular risk. Epidemiology.

[4] de Vries ST, Denig P, Ekhart C, Burgers JS, Kleefstra N, Mol PG, van Puijenbroek EP (2019). Sex differences in adverse drug reactions reported to the National Pharmacovigilance Centre in the Netherlands: An explorative observational study. Br J Clin Pharmacol.

[5] Kongkaew C, Noyce PR, Ashcroft DM (2008). Hospital admissions associated with adverse drug reactions: a systematic review of prospective observational studies. Ann Pharmacother.

[6] Sultana J, Cutroneo P, Trifirò G (2013). Clinical and economic burden of adverse drug reactions. J Pharmacol Pharmacother.

[7] Postigo R, Brosch S, Slattery J, van Haren A, Dogné J, Kurz X, Candore G, Domergue F, Arlett P (2018). EudraVigilance medicines safety database: publicly accessible data for research and public health protection. Drug Saf.

[8] Murdoch TB, Detsky AS (2013). The inevitable application of big data to health care. J Am Med Assoc.

[9] Sheikhalishahi S, Miotto R, Dudley JT, Lavelli A, Rinaldi F, Osmani V (2019). Natural language processing of clinical notes on chronic diseases: systematic review. JMIR Med Inform.

[10] Juhn Y, Liu H (2020). Artificial intelligence approaches using natural language processing to advance EHR-based clinical research. J Allergy Clin Immunol.

[11] Khattak FK, Jeblee S, Pou-Prom C, Abdalla M, Meaney C, Rudzicz F (2019). A survey of word embeddings for clinical text. J Biomed Inform.

[12] Zhao M, Masino A, Yang C (2018). A framework for developing and evaluating word embeddings of drug-named entity. Proceedings of the BioNLP 2018 workshop.

[13] Wang Y, Liu S, Afzal N, Rastegar-Mojarad M, Wang L, Shen F, Kingsbury P, Liu H (2018). A comparison of word embeddings for the biomedical natural language processing. J Biomed Inform.

[14] Banerjee I, Madhavan S, Goldman R, Rubin D (2017). Intelligent word embeddings of free-text radiology reports. arXiv.

[15] Sammani A, Bagheri A, van der Heijden PG, Te Riele AS, Baas AF, Oosters CA, Oberski D, Asselbergs FW (2021). Automatic multilabel detection of ICD10 codes in Dutch cardiology discharge letters using neural networks. NPJ Digit Med.

[16] Dai H, Su C, Wu C (2020). Adverse drug event and medication extraction in electronic health records via a cascading architecture with different sequence labeling models and word embeddings. J Am Med Inform Assoc.

[17] Brown EG, Wood L, Wood S (1999). The medical dictionary for regulatory activities (MedDRA). Drug Saf.

[18] Bots SH, Siegersma KR, Onland-Moret NC, Asselbergs FW, Somsen GA, Tulevski II, den Ruijter HM, Hofstra L (2021). Routine clinical care data from thirteen cardiac outpatient clinics: design of the Cardiology Centers of the Netherlands (CCN) database. BMC Cardiovasc Disord.

[19] Menger V, Scheepers F, van Wijk LM, Spruit M (2018). DEDUCE: a pattern matching method for automatic de-identification of Dutch medical text. Telemat Informat.

[20] Sproat R, Black AW, Chen S, Kumar S, Ostendorf M, Richards C (2001). Normalization of non-standard words. Comput Speech Lang.

[21] Python Language Reference, Version 3.7.9.

[22] Bird S (2006). NLTK: The Natural Language Toolkit. Proceedings of the COLING/ACL on Interactive Presentation Sessions.

[23] Rehurek R, Sojka P (2010). Software framework for topic modelling with large corpora. Proceedings of LREC 2010 workshop New Challenges for NLP Frameworks.

[24] Mikolov T, Chen K, Corrado G, Dean J (2013). Efficient estimation of word representations in vector space. Proceedings of the 1st International Conference on Learning Representations, ICLR 2013.

[25] Wang B, Wang A, Chen F, Wang Y, Kuo CJ (2019). Evaluating word embedding models: methods and experimental results. APSIPA Transactions on Signal and Information Processing.

[26] R Core Team (2020). R: A language and environment for statistical computing. R Foundation for Statistical Computing, Vienna, Austria.

[27] RStudio Team (2021). RStudio: integrated development environment for R. RStudio, PBC, Boston, MA.

[28] Kuhn M (2009). The caret Package.

[29] Honigman B, Lee J, Rothschild J, Light P, Pulling RM, Yu T, Bates DW (2001). Using computerized data to identify adverse drug events in outpatients. J Am Med Inform Assoc.

[30] Murff Harvey J, Forster Alan J, Peterson Josh F, Fiskio Julie M, Heiman Heather L, Bates David W (2003). Electronically screening discharge summaries for adverse medical events. J Am Med Inform Assoc.

[31] Cantor MN, Feldman HJ, Triola MM (2007). Using trigger phrases to detect adverse drug reactions in ambulatory care notes. Qual Saf Health Care.

[32] Eriksson R, Jensen PB, Frankild S, Jensen LJ, Brunak S (2013). Dictionary construction and identification of possible adverse drug events in Danish clinical narrative text. J Am Med Inform Assoc.

[33] Tang Y, Yang J, Ang PS, Dorajoo SR, Foo B, Soh S, Tan SH, Tham MY, Ye Q, Shek L, Sung C, Tung A (2019). Detecting adverse drug reactions in discharge summaries of electronic medical records using Readpeer. Int J Med Inform.

[34] Abdalla Mohamed, Abdalla Moustafa, Rudzicz F, Hirst G (2020). Using word embeddings to improve the privacy of clinical notes. J Am Med Inform Assoc.

[35] Iqbal E, Mallah R, Jackson RG, Ball M, Ibrahim ZM, Broadbent M, Dzahini O, Stewart R, Johnston C, Dobson RJ (2015). Identification of adverse drug events from free text electronic patient records and information in a large mental health case register. PLoS One.

[36] Névéol A, Dalianis H, Velupillai S, Savova G, Zweigenbaum P (2018). Clinical natural language processing in languages other than English: opportunities and challenges. J Biomed Semantics.

