# Supplementary material for: Development of a Pipeline for Adverse Drug Reaction Identification in Clinical Notes: Word Embedding Models and String Matching
Source: JMIR Med Inform. 2022 Jan 25;10(1):e31063. doi: 10.2196/31063 (PMC8826143; doi:10.2196/31063)
Supplement: Multimedia Appendix 1 [file medinform_v10i1e31063_app1.docx]

#
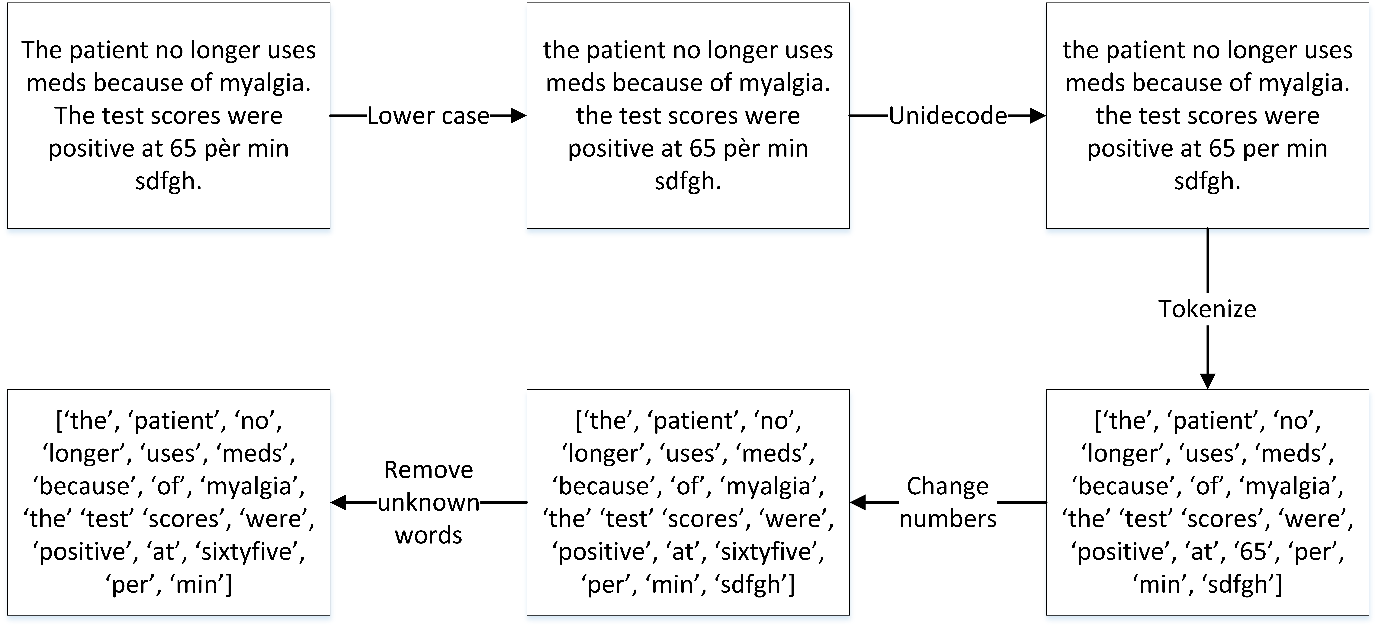
Multimedia Appendix 1 Supplementary methods: text preprocessing and threshold setting for word embedding models

**Figure S1.** Example of pre-processing performed on the clinical notes.

## Cosine similarity in word embedding models


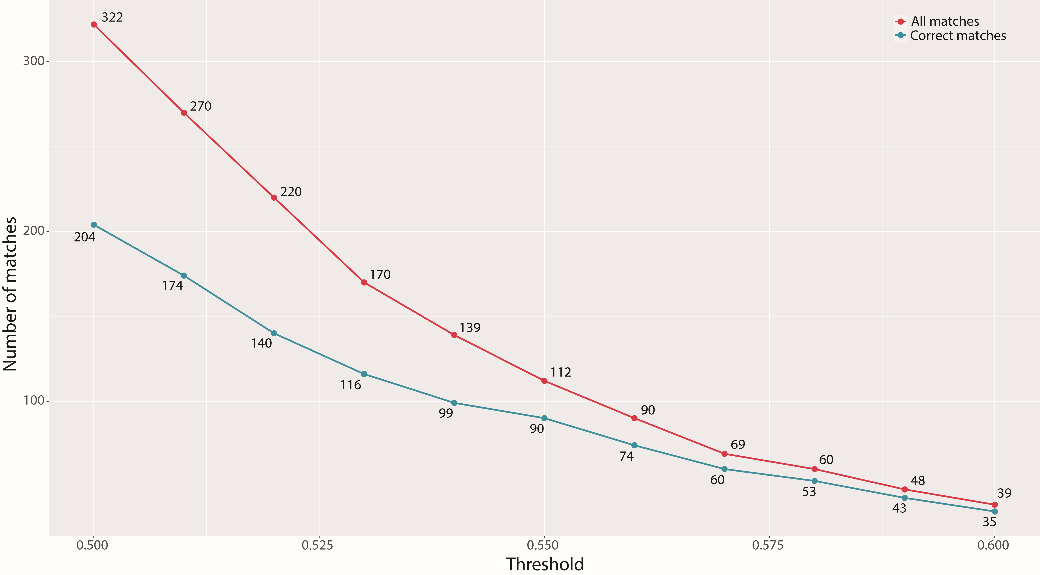
The cosine similarity between search words and the words in the search area of the clinical note was used to extract medications and ADRs from text. Thresholds for cosine similarity were defined based on the trade-off between the number of identified ADRs and correct matches. This analysis was performed on the validation set. For each search word, a grid search was performed with thresholds between 0.50 and 0.60 to determine the optimal threshold value. A threshold was determined in a way that it captures a significant number of words, without generating too many false positives and are based on visual inspection of figures such as Multimedia Appendix 1, figure S2 and evaluation of the most similar words as is displayed for a specific selection in Table 3. False positives are generated when the threshold is set at a value that is too low. Furthermore, different search words had overlap in their identified matches. Therefore, the chosen threshold could be set to a higher value, because another synonym of the word is already captured by another search word. This reduces the number of false positives. An example of threshold evaluation is shown in Supplementary figure 1 for the word ‘dizziness. The selected threshold was set to 0.58.

**Figure S2.** Grid search to evaluate the threshold for the search word 'dizziness'
