# Supplementary material for: Development of a Pipeline for Adverse Drug Reaction Identification in Clinical Notes: Word Embedding Models and String Matching
Source: JMIR Med Inform. 2022 Jan 25;10(1):e31063. doi: 10.2196/31063 (PMC8826143; doi:10.2196/31063)
Supplement: Multimedia Appendix 2 [file medinform_v10i1e31063_app2.docx]

# Multimedia Appendix 2 Overview of model settings and results

**Table S1.** Search words - Dutch and (English) for the identification of ADRs, medication and the predefined cosine similarity threshold.

| **ADR^a^ Search Words** | **Threshold** | **Medication Search Words** | **Threshold** |
| --- | --- | --- | --- |
| Myalgie (myalgia) | 0.50 | Acenocoumarol (Acenocoumarol) | 0.68 |
| Nierfunctiestoornis (kidney dysfunction) | 0.60 | Amiodarone (Amiodaron) | 0.64 |
| Kriebelhoest (cough) | 0.55 | Amlodipine (Amlodipine) | 0.67 |
| Oedeem (edema) | 0.60 | Aspirine (Aspirin) | 0.65 |
| Hypotensie (hypotension) | 0.57 | Clopidogrel (Clopidogrel) | 0.63 |
| Aritmie (arrythmia) | 0.50 | Gemfibrozil (Gemfibrozil) | 0.76 |
| Moeheid (fatigue) | 0.55 | Hydrochloorthiazide (Hydrochlorothiazide) | 0.66 |
| Duizeligheid (dizzyness) | 0.58 | Medicatie (Medication) | 0.61 |
| Hoofdpijn (headache) | 0.56 | Metoprolol (Metoprolol)  Beta (beta) | 0.70  0.63 |
| Hematome (haematoma) | 0.54 | Nitroglycerine (Nitroglycerin) | 0.65 |
| Atriumfibrileren (atrial fibrillation) | 0.59 | Perindopril (Perindopril) | 0.68 |
| pijn_op_de_borst (chest pain) | 0.60 | Statine (Statin) | 0.66 |
| lage_rr (low blood pressure) | 0.51 | Tamsulosine (Tamsolusin) | 0.75 |
| verminderde_conditie (decreased stamina) | 0.60 | Valsartan (Valsartan) | 0.67 |
| dikke_enkels (swollen ankles) | 0.60 |  |  |
| hoge_bloeddruk (high blood pressure) | 0.58 |  |  |
| hoge_hr (high heart rate) | 0.58 |  |  |
| chonotrope_incompetentie (chronotropic incompetence) | 0.70 |  |  |
| traag_sinusritme (slow sinusrhythm) | 0.77 |  |  |
| qrs_verbreding (qrs elongation) | 0.72 |  |  |
| depressieve_gevoelens (depressed feelings) | 0.75 |  |  |
| laag_kalium (low potassium) | 0.65 |  |  |
| pijnlijk_gevoel (painful feelings) | 0.80 |  |  |

^a^ADR: adverse drug reaction

**Table S2.** Frequency of different ADRs manually extracted from the free text of the reason for discontinuation of a prescription.

| **ADR^a^** | **Number of mentions (%)** |
| --- | --- |
| Myalgia | 157 |
| Oedema | 84 |
| Kidney Dysfunction | 72 |
| Cough | 67 |
| Arrhythmia | 49 |
| Hypotension | 48 |
| Fatigue | 48 |
| Dizziness | 44 |
| Headache | 30 |
| Stomach pain | 27 |

^a^ADR: adverse drug reaction

**Table S3.** Words that indicate a change or the initiation of medication. These words stop further evaluation of ADRs and medication in the text.

| **Not-ADR^a^ keywords before Medication** | **Not-ADR keywords after medication** |
| --- | --- |
| Verdubbeling (double) | Verhoogd (increased) |
| Verhoogde (increased) | Verdubbeld (doubled) |
| Dosisverhoging (increased dose) | Verdubbeling (double) |
| Begonnen (started) | Gestart (started) |
| Start (start) | Verhogen (to increase) |
| Ophogen (increased) | Opgehoogd (increased) |
| Hoogde (increased) | Uitgebreid (expanded) |
| Hoog (increase) | Toegevoegd (Added) |
|  | Begonnen (started) |
|  | Ophogen (to increase) |
|  | Hoogde (increased) |

^a^ADR: adverse drug reaction

| **Version** | **Task** | **TN**^a^ | **FN^b^** | **FP^c^** | **TP^d^** |
| --- | --- | --- | --- | --- | --- |
| version_1A | Binary | 639 | 72 | 112 | 165 |
| version_1A | ADR^e^ | 639 | 174 | 270 | 190 |
| version_1A | Medication | 639 | 118 | 256 | 168 |
| version_1A | ADR and Medication | 639 | 239 | 491 | 153 |
| version_1B | Binary | 664 | 79 | 87 | 158 |
| version_1B | ADR | 664 | 186 | 191 | 178 |
| version_1B | Medication | 664 | 125 | 209 | 161 |
| version_1B | ADR and Medication | 664 | 247 | 378 | 145 |
| version_2A | Binary | 531 | 67 | 220 | 170 |
| version_2A | ADR | 531 | 156 | 828 | 208 |
| version_2A | Medication | 531 | 118 | 562 | 168 |
| version_2A | ADR and Medication | 531 | 237 | 2028 | 155 |
| version_2B | Binary | 566 | 73 | 185 | 164 |
| version_2B | ADR | 566 | 167 | 526 | 197 |
| version_2B | Medication | 566 | 126 | 498 | 160 |
| version_2B | ADR and Medication | 566 | 250 | 1417 | 142 |
| version_3A | Binary | 651 | 74 | 100 | 163 |
| version_3A | ADR | 651 | 171 | 223 | 193 |
| version_3A | Medication | 651 | 121 | 211 | 165 |
| version_3A | ADR and Medication | 651 | 240 | 391 | 152 |
| version_3B | Binary | 676 | 80 | 75 | 157 |
| version_3B | ADR | 676 | 183 | 157 | 181 |
| version_3B | Medication | 676 | 128 | 163 | 158 |
| version_3B | ADR and Medication | 676 | 248 | 298 | 144 |
| version_4A | Binary | 576 | 53 | 175 | 184 |
| version_4A | ADR | 576 | 147 | 432 | 217 |
| version_4A | Medication | 576 | 103 | 375 | 183 |
| version_4A | ADR and Medication | 576 | 223 | 778 | 169 |
| version_4B | Binary | 614 | 63 | 137 | 174 |
| version_4B | ADR | 614 | 159 | 296 | 205 |
| version_4B | Medication | 614 | 114 | 295 | 172 |
| version_4B | ADR and Medication | 614 | 235 | 581 | 157 |
| version_5A | Binary | 678 | 98 | 73 | 139 |
| version_5A | ADR | 678 | 217 | 149 | 147 |
| version_5A | Medication | 678 | 154 | 145 | 132 |
| version_5A | ADR and Medication | 678 | 273 | 235 | 119 |
| version_5B | Binary | 699 | 112 | 52 | 125 |
| version_5B | ADR | 699 | 226 | 99 | 138 |
| version_5B | Medication | 699 | 163 | 108 | 123 |
| version_5B | ADR and Medication | 699 | 279 | 173 | 113 |
| version_6A | Binary | 641 | 82 | 110 | 155 |
| version_6A | ADR | 641 | 203 | 237 | 161 |
| version_6A | Medication | 641 | 139 | 220 | 147 |
| version_6A | ADR and Medication | 641 | 265 | 367 | 127 |
| version_6B | Binary | 669 | 99 | 82 | 138 |
| version_6B | ADR | 669 | 213 | 153 | 151 |
| version_6B | Medication | 669 | 151 | 160 | 135 |
| version_6B | ADR and Medication | 669 | 273 | 261 | 119 |

**Table S4.** Overview of confusion matrix for all versions of the pipeline and all tasks.

^a^TN: true negatives, ^b^FN: false negatives, ^c^FP: false positives, ^d‑^TP: true positives, ^e^ADR: adverse drug reaction
