# Supplementary material for: Development of a Pipeline for Adverse Drug Reaction Identification in Clinical Notes: Word Embedding Models and String Matching
Source: JMIR Med Inform. 2022 Jan 25;10(1):e31063. doi: 10.2196/31063 (PMC8826143; doi:10.2196/31063)
Supplement: Multimedia Appendix 3 [file medinform_v10i1e31063_app3.docx]

# Multimedia Appendix 3 Evaluation of Pipeline

The different versions of the pipeline are evaluated on different tasks. To evaluate the performance of each version of the pipeline on different tasks, different metrics and their calculations are shown in Table S1. Table S2 gives four different examples of the result of manual labeling and the result of a version of the pipeline. In Table S3, Table S4, Table S5, and Table S6, the number of true positives (TN), false positives (FP), true negatives (TN) and false negatives (FN) are shown per example.

**Table S1.** Overview of used metrics

| **Metrics** | **Calculation** | **Explanation** |
| --- | --- | --- |
| *Accuracy* | (TP^a^ + TN^b^)/(TN + TP + FN^c^ + FP^d^) | Proportion of samples that have been correctly classified. |
| *Sensitivity or Recall* | TP/(TP + FN) | Proportion of true positives that are classified correctly, given the number of actual positives. |
| *Specificity* | TN/(TN + FP) | Proportion of true negatives that are classified correctly, given the number of actual negatives. |
| *Precision or PPV^e^* | TP/(TP + FP) | Proportion of true positives that are classified correctly, given the number of positive classifications. |
| *NPV^f^* | TN/(TN + FN) | Proportion of true negatives that are classified correctly, given the number of negative classifications. |
| *F1-score* | 2*((precision*recall)/(precision + recall)) | Balance between precision and recall. |
| *Detection rate* | TP/(TN + FP + FN + FP) | Proportion of classifications that are correctly positively classified. |
| *Detection prevalence* | (TN + FP)/(TN + TP + FN + FP) | Proportion of classifications that are positively classified |
| *Balanced accuracy* | (Sensitivity + Specificity)/2 | Average of sensitivity and specificity. |

^a^TP: true positives, ^b^TN: true negatives, ^c^FN: false negatives, ^d‑^FP: false positives, ^e^PPV: positive predictive value, ^f^NPV: negative predictive value.

**Table S2.** Four examples of consult notes, the result of manual labeling and a possible result of the pipeline.

| **Text** | **Manual labeling** | **Manual label** | **Result Pipeline** | **Pipeline label** |
| --- | --- | --- | --- | --- |
| Patient has developed evident hypertension with signs of clvh/strain, c.q. diastolic lv dysfunction. On the other hand, no signs for increased r pressure or dilated r structures. Pat was convinced to change antihypertensive medication. | [] | 0 | [] | 0 |
| This is a patient with elaborate cardiac history, at this moment hypertension, ace inhibitor was stopped. Besides, statin was halved. | [] | 0 | [ace, hypertension], [inhibitor, hypertension] | 1 |
| <Person> because hefty rectal bleeding with known colitis. Ufn stop ascal. | [ascal, rectal bleeding] | 1 | [] | 0 |
| Monitoring after ablation, no complaints (probably atrial fibrillation once, has lost more than 10 kilograms) is doing well. Echocardiogram and stress test did nothing details. Decrease metoprolol. Control 3 months. C/ state after uncomplicated isolation of lung veins icw atrial fibrillation without complaints amiodaron decreased normal stress ecg normal echocardiogram hypertriglyceridaemia b/ decrease metoprolol wrt hypertriglyceridaemia (temporarily) stop statin/start modalim co 3 months | [statin, hypertriglyceridaemia] | 1 | [metoprolol, hypertriglyceridaemia], [metoprolol, atrial fibrilation] | 1 |

## Task A: Binary Evaluation

**Table S3.** Four examples of consult notes and the evaluation of binary labeling for the presence or absence of ADR information.

| **Text** | **Manual label** | **Pipeline label** | **Class** |
| --- | --- | --- | --- |
| Patient has developed evident hypertension with signs of clvh/strain, c.q. diastolic lv dysfunction. On the other hand, no signs for increased r pressure or dilated r structures. Pat was convinced to change antihypertensive medication. | 0 | 0 | TN |
| This is a patient with elaborate cardiac history, at this moment hypertension, ace inhibitor was stopped. Besides, statin was halved. | 0 | 1 | FP |
| <Person> because hefty rectal bleeding with known colitis. Ufn stop ascal. | 1 | 0 | FN |
| Monitoring after ablation, no complaints (probably atrial fibrillation once, has lost more than 10 kilograms) is doing well. Echocardiogram and stress test did nothing details. Decrease metoprolol. Control 3 months. C/ state after uncomplicated isolation of lung veins icw atrial fibrillation without complaints amiodaron decreased normal stress ecg normal echocardiogram hypertriglyceridaemia b/ decrease metoprolol wrt hypertriglyceridaemia (temporarily) stop statin/start modalim co 3 months | 1 | 1 | TN |

## Task B: Medication Evaluation

**Table S5.** Four examples of consult notes and the evaluation of the outcome of the pipeline on the extraction of medication that caused the ADR.

| **Text** | **Manual labeling** | **Result Pipeline** | **Class** |
| --- | --- | --- | --- |
| Patient has developed evident hypertension with signs of clvh/strain, c.q. diastolic lv dysfunction. On the other hand, no signs for increased r pressure or dilated r structures. Pat was convinced to change antihypertensive medication. | [] | [] | TN |
| This is a patient with elaborate cardiac history, at this moment hypertension, ace inhibitor was stopped. Besides, statin was halved. | [] | [ace], [inhibitor] | FP (2) |
| <Person> because hefty rectal bleeding with known colitis. Ufn stop ascal. | [ascal] | [] | FN (1) |
| Monitoring after ablation, no complaints (probably atrial fibrillation once, has lost more than 10 kilograms) is doing well. Echocardiogram and stress test did nothing details. Decrease metoprolol. Control 3 months. C/ state after uncomplicated isolation of lung veins icw atrial fibrillation without complaints amiodaron decreased normal stress ecg normal echocardiogram hypertriglyceridaemia b/ decrease metoprolol wrt hypertriglyceridaemia (temporarily) stop statin/start modalim co 3 months | [statin] | [metoprolol] | FP (1)  FN (1) |

## Task C: Evaluation of ADR

**Table S4.** Four examples of consult notes and the evaluation of the outcome of the pipeline on the extraction of ADRs.

| **Text** | **Manual labeling** | **Result Pipeline** | **Class** |
| --- | --- | --- | --- |
| Patient has developed evident hypertension with signs of clvh/strain, c.q. diastolic lv dysfunction. On the other hand, no signs for increased r pressure or dilated r structures. Pat was convinced to change antihypertensive medication. | [] | [] | TN |
| This is a patient with elaborate cardiac history, at this moment hypertension, ace inhibitor was stopped. Besides, statin was halved. | [] | [hypertension], [hypertension] | FP (1, because 1 unique ADR) |
| <Person> because hefty rectal bleeding with known colitis. Ufn stop ascal. | [rectal bleeding] | [] | FN (1) |
| Monitoring after ablation, no complaints (probably atrial fibrillation once, has lost more than 10 kilograms) is doing well. Echocardiogram and stress test did nothing details. Decrease metoprolol. Control 3 months. C/ state after uncomplicated isolation of lung veins icw atrial fibrillation without complaints amiodaron decreased normal stress ecg normal echocardiogram hypertriglyceridaemia b/ decrease metoprolol wrt hypertriglyceridaemia (temporarily) stop statin/start modalim co 3 months | [hypertriglyceridaemia] | [hypertriglyceridaemia], [atrial fibrilation] | TP (1)  FP (1) |

## Task D: Evaluation of Medication and ADR

**Table S6.** Four examples of consult notes and the evaluation of the outcome of the pipeline on the extraction of the combination of medication and ADR.

| **Text** | **Manual labeling** | **Result Pipeline** | **Class** |
| --- | --- | --- | --- |
| Patient has developed evident hypertension with signs of clvh/strain, c.q. diastolic lv dysfunction. On the other hand, no signs for increased r pressure or dilated r structures. Pat was convinced to change antihypertensive medication. | [] | [] | TN |
| This is a patient with elaborate cardiac history, at this moment hypertension, ace inhibitor was stopped. Besides, statin was halved. | [] | [ace, hypertension], [inhibitor, hypertension] | FP (2) |
| <Person> because hefty rectal bleeding with known colitis. Ufn stop ascal. | [ascal, rectal bleeding] | [] | FN (1) |
| Monitoring after ablation, no complaints (probably atrial fibrillation once, has lost more than 10 kilograms) is doing well. Echocardiogram and stress test did nothing details. Decrease metoprolol. Control 3 months. C/ state after uncomplicated isolation of lung veins icw atrial fibrillation without complaints amiodaron decreased normal stress ecg normal echocardiogram hypertriglyceridaemia b/ decrease metoprolol wrt hypertriglyceridaemia (temporarily) stop statin/start modalim co 3 months | [statin, hypertriglyceridaemia] | [metoprolol, hypertriglyceridaemia], [metoprolol, atrial fibrilation] | FP (2)  FN (1) |
